# *Muscodor albus* Volatiles Control Toxigenic Fungi under Controlled Atmosphere (CA) Storage Conditions

**DOI:** 10.3390/ijms131215848

**Published:** 2012-11-27

**Authors:** Gordon Braun, Matteo Vailati, Robert Prange, Eric Bevis

**Affiliations:** 1 Atlantic Food and Horticulture Research Centre, Agriculture and Agri-Food Canada, 32 Main St., Kentville, NS B4N 1J5, Canada; E-Mail: eric.bevis@agr.gc.ca; 2 Via Farense n.91, Passo Corese, 02032 Fara in Sabina (RI), Italy; E-Mail: mat.vai@libero.it; 3 Faculty of Agriculture, Dalhousie University, Truro, NS B2N 5E3, Canada; E-Mail: rkprange@gmail.com

**Keywords:** *Muscodor albus*, post-harvest pathogen control, controlled atmosphere storage, toxigenic fungi, mycotoxin, fumigation, natural volatiles

## Abstract

*Muscodor albus*, a biofumigant fungus, has the potential to control post-harvest pathogens in storage. It has been shown to produce over 20 volatile compounds with fungicidal, bactericidal and insecticidal properties. However, *M. albus* is a warm climate endophyte, and its biofumigant activity is significantly inhibited at temperatures below 5 °C. Conidia of seven mycotoxin producing fungi, *Aspergillus carbonarius*, *A. flavus*, *A. niger*, *A. ochraceus*, *Penicillium verrucosum*, *Fusarium culmorum* and *F. graminearum*, were killed or prevented from germinating by exposure to volatiles from 2 g *M. albus*-colonized rye grain per L of headspace in sealed glass jars for 24 h at 20 °C. Two major volatiles of *M. albus*, isobutyric acid (IBA) and 2-methyl-1-butanol (2MB) at 50 μL/L and 100 μL/L, respectively, gave differential control of the seven fungi when applied individually at 20 °C. When the fungi were exposed to both IBA and 2MB together, an average of 94% of the conidia were killed or suppressed. In a factorial experiment with controlled atmosphere storage (CA) at 3 °C and 72 h exposure to four concentrations of IBA and 2MB combinations, 50 μL/L IBA plus 100 μL/L 2MB killed or suppressed germination of the conidia of all seven fungi. Controlled atmosphere had no significant effect on conidial viability or volatile efficacy. Major volatiles of *M. albus* may have significant potential to control plant pathogens in either ambient air or CA storage at temperatures below 5 °C. However, combinations of volatiles may be required to provide a broader spectrum of control than individual volatiles.

## 1. Introduction

The discovery of *Muscodor albus* Worapong, Strobel & W.M.Hess, an endophyte of the cinnamon tree (*Cinnamomum zeylanicum* Blume.), introduced a new potential biocontrol for storage rots of fruits [1–3]. *M. albus*, like some other fungi, produces a number of volatile organic compounds that have biocidal or biostatic activity against a variety of fungal and bacterial pathogens [3–5]. Just a few hours of rehydration of rye seeds colonized by *M. albus* results in the production of various organic volatiles. In some instances, a 24 h exposure at 20 °C to the reactivated *M. albus* is sufficient to kill or suppress fungal conidia and bacteria [1,6,7]. However, many fruits are picked and cooled immediately to between 0 and 5 °C to increase the length of storage before fruit quality is compromised by bacterial or fungal pathogens or physiological breakdown. *M. albus* is a tropical endophyte and demonstrates no growth and little antifungal or antibacterial activity at temperatures below 5 °C [5]. This characteristic significantly compromises the utility of *M. albus* as a biocontrol for many stored fruit products. To overcome this problem, larger quantities of *M. albus* rye seed can be pre-activated by dipping in water 2–6 h before placing in cartons of fruit entering cold storage [8–10]. The purpose of this study was to determine if the main *M. albus* volatiles, isobutyric acid and 2-methyl-1-butanol in the absence of the fungus, could effectively control the toxigenic fungi, *Aspergillus, flavus* Link, *A. niger* Tiegh., *A. carbonarius* (Bainier) Thom, *A. ochraceus* G. Wilh., *Penicillium verrucosum* Dierckx, *Fusarium graminearum* Schwabe and *F. culmorum* (W.G. Sm.) Sacc., at temperatures below 5 °C and with controlled atmospheres. These fungi are responsible for contaminating various fruits, nuts and cereals with mycotoxins [11].

## 2. Results and Discussion

### 2.1. *Muscodor albus* Efficacy

Not all pathogens tested responded equally to the natural volatiles produced by *M. albus* (Table 1). On average, *P. verrucosum* and *F. graminearum* conidia were more tolerant of *M. albus* volatiles than the other fungal species. Also, 88% of the conidia were killed or prevented from germinating by 0.5 g or greater of *M. albus* colonized rye seed per liter of storage volume. The conidia of all seven of the fungal species tested were killed or suppressed by a 24 h exposure to the volatiles produced by 2 g/L of *M. albus*. There was a significant pathogen species × *M. albus* rate interaction, indicating that not all the toxigenic fungi tested were equally sensitive to the *M. albus* volatiles.

### 2.2. Efficacy of Two *M. albus* Volatiles

The two *M. albus* volatiles, isobutyric acid and 2-methyl-1-butanol, applied singly, behaved as expected for fungal toxicants (Table 2). Response curves of fungal species to increasing concentrations of IBA or 2MB are sigmoidal or logistic growth curves and, therefore, not linear. Increasing the concentration or exposure time of the fungal conidia to the volatiles increased the proportion (dead or non-germinated conidia/total No. of conidia) of conidia killed or suppressed (main effects). In addition, there was a significant interaction between the fungal species, type and concentration of volatile. For example, *F. graminearum* and *F. culmorum* conidia were completely killed or germination suppressed by IBA at 37.5 μL/L, while 2MB killed/suppressed 26% or less of the conidia at more than twice the concentration (100 μL/L). Conversely, *A. flavus* and *A. niger* were poorly controlled by IBA at the highest concentration, but were better controlled by the highest rate of 2MB. *A. carbonarius* and *P. verrucosum* were equally well-controlled by the highest rate of both volatiles, while *A. ochraceus* was poorly controlled by either volatile. Clearly, even species within the same genus have different sensitivities to the toxic effect of the *M. albus* volatiles tested in this study.

To clarify some confusion in the published literature on the identity of one volatile (1,3), this experiment was repeated with 3-methyl-1-butanol (3MB) replacing 2MB. It resulted in no significant difference (*p* = 0.05) in control between 3MB and 2MB (data not shown). These compounds appear to be equal in their activity against conidial germination for the fungi tested.

### 2.3. Efficacy of *M. albus* Volatile Mixtures

Mixtures of IBA and 2MB killed/suppressed a significant number of conidia of all seven toxigenic fungi tested (Table 3). The highest concentration of the volatile mixture had the greatest killing/suppression effect with complete control of *A. carbonarius*, *A. ochraceus*, *P. verrucosum* and *F. culmorum* and a greater than 80% kill/suppression of the remaining fungi. Interestingly, in the previous experiment, *A. ochraceus* was poorly controlled by both volatiles individually (Table 2), but a mixture killed/suppressed all the conidia and had a synergism ratio of 1.25, indicative of a synergistic effect [12]. However, the effect of the volatile combinations on the other fungal species was consistent with an additive effect with synergism ratios of 1 ± 0.05.

### 2.4. Efficacy of Volatile Mixtures under CA Storage Conditions

**Figure 1:**
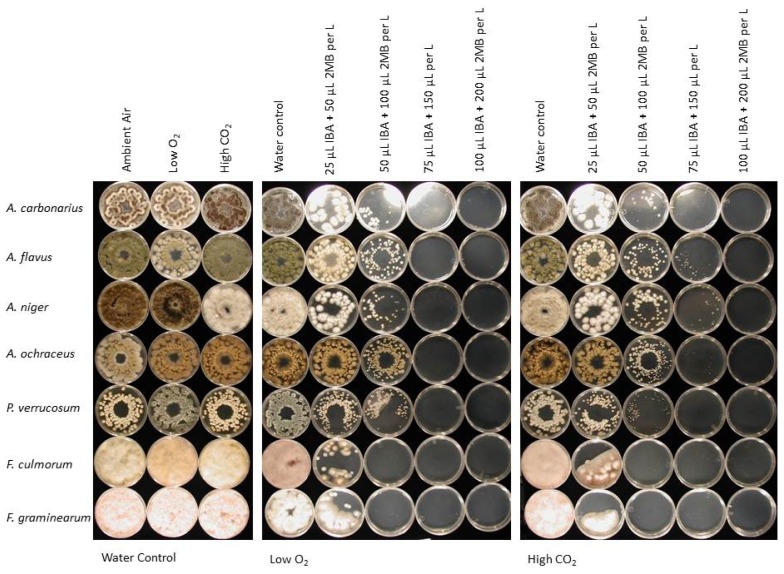
Conidial germination and fungal growth of mycotoxin-producing *Aspergillus*, *Penicillium* and *Fusarium* species on potato dextrose agar exposed to low oxygen (1% O_2_ and 0.03% CO_2_) or high CO_2_ (20.8% O_2_ and 15% CO_2_) atmospheres at 3 °C for 72 h in the presence of four concentrations of isobutyric acid (IBA) and 2-methyl-1-butanol (2MB)(25 + 50, 50 + 100, 75 + 150 or 100 + 200 μL/L of container headspace). The treated cultures were incubated in ambient air at room temperature for seven days after treatment and then photographed.

Both fungal species and concentration of the volatile mixture had a significant effect on the proportion of conidia killed/suppressed under CA storage conditions (Table 4, Figure 1). There was a significantly different response of the fungi tested with a range of control averaging from 28% to 49% over all the volatile concentrations tested. Interestingly, at the highest concentration, 100% of the conidia of all the toxigenic fungi were killed/suppressed at 3 °C. Statistical analysis of conidial germination demonstrated that the low oxygen or high carbon dioxide atmospheres had no significant effect on germination. Therefore, the *M. albus* volatiles tested in this study have been shown to effectively kill/suppress the conidia of seven toxigenic fungi at temperatures down to 3 °C, regardless of storage atmospheres used in this study. Our previous work had demonstrated that reactivated *M. albus*-colonized rye seed is not very effective in controlling bacteria and fungi at temperatures below 5 °C [6], thus limiting its application in cold and CA storage. However, this study has demonstrated that a mixture of two of the main *M. albus* volatiles in the absence of the fungus was effective in killing/suppressing fungal conidia under CA storage conditions.

## 3. Experimental Section

Dry, *M. albus* strain 620-colonized rye seed (from J. Mercier, AgraQuest Inc., Davis, CA, USA) was stored at 4 °C and warmed to room temperature for 2 h before being used in experiments. The major *M. albus* volatiles, isobutyric acid, 2-methyl-1-butanol and 3-methyl-1-butanol, were purchased from Sigma-Aldrich (Oakville, ON, Canada).

**Figure 2:**
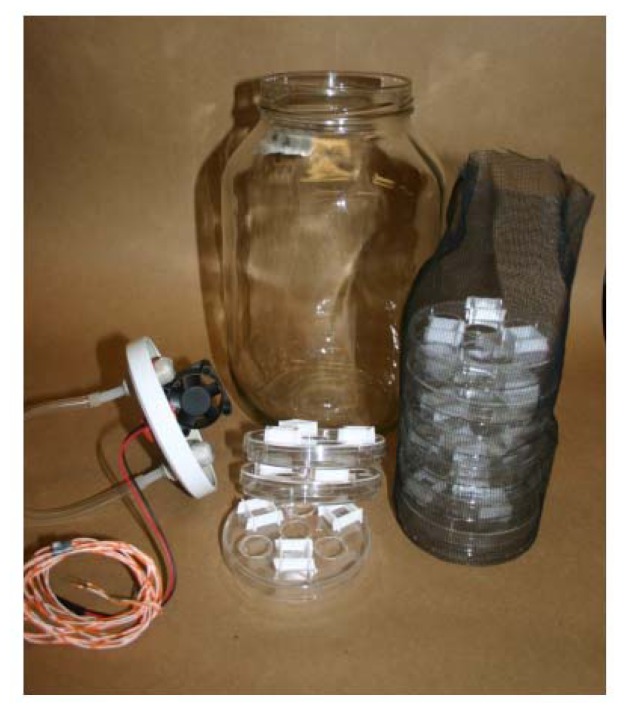
Four liter jar, lid with fan and nylon mesh sleeve with Petri dishes and perforated lids with spacers used to incubate PDA plates of fungal conidia with *M. albus* volatiles.

Fungal isolate *F. graminearum* (DAOM 232357), *F. culmorum* (DAOM 232357), *A. flavus* (DAOM 222004), *A. niger* (DAOM 222006), *A. ochraceus* (DAOM 22007) and *P. verrucosum* (DAOM) 213266) were obtained from the Canadian Collection of Fungus Cultures (Ottawa, ON, Canada). *A. carbonarius* (ATCC 201259) was purchased from the American Type Culture Collection (Manassas, VA, USA). Macroconidia of *Fusarium* spp. were produced on SNA media to which 0.5 cm squares of sterile filter paper had been adhered to induce sporulation [13]. Cultures were grown at a constant 27 °C with 12-h day/night cycles. Light was provided by one UV fluorescent bulb (GE F15T8.BLB) and two white light fluorescent bulbs (GE F15T12.KB). All other fungi were grown at 27 °C in the dark. Conidia were harvested by flooding cultures with sterile distilled water (SDW) and dislodging them with a bent glass rod. In all experiments in this study, conidial suspensions were diluted with SDW to give an absorbance reading of 0.05 units at 600 nm. One mL of these conidial suspensions was transferred to 99 mL of SDW and 0.0492 mL applied to 10 cm Petri dishes of potato dextrose agar (PDA) with a Spiral Plater™ (Spiral Biotech., Bethesda, MD, USA). One dish of each fungal species of freshly-seeded conidia (~100 conidia/dish) on PDA, capped with a porous lid and a 0.5 cm spacer to allow air circulation, were stacked in no specific order in a nylon mesh sleeve and placed into a 4 L jar [7] (Figure 2). On top of each stack of Petri dishes was placed a glass Petri dish lid containing Whatman filter paper #1 and dry *M. albus*-colonized rye seed or the major *M. albus* volatiles. The jars were capped with a lid with a 40 mm × 40 mm × 20 mm fan (12 VDC, 5.2 ft^3^/min capacity; Mode Electronics, Burnaby, BC, Canada) attached to the inside surface and a rubber septum sealing a hole (wiring port) in the top of the lid. The lid/jar interface and the rubber septum/wiring port were all sealed air-tight with putty. The fans were turned on to maximum speed to move the volatiles throughout the contents of the jars. The jars were incubated for 24 h at 20 °C in a controlled environment chamber. The jars were then opened and the stacks of Petri dishes removed from the jars in a laminar flow hood and left for 10 min to dissipate any residual volatiles. Then, normal Petri dish lids replaced the perforated lids, and the treated dishes of fungal conidia were incubated for six days in an incubator at 20 °C. The total duration of conidial incubation was seven days, unless stated otherwise. The number of conidia that did not germinate (dead/suppressed) out of ~100 conidia counted under a microscope was recorded each day for five days during the incubation period. The Petri dishes from the experiment were incubated for another seven days and checked to verify that fungal growth did not resume from conidia considered dead/suppressed. All experiments were conducted twice.

### 3.1. *M. albus* Efficacy

In the first experiment, 0, 0.5, 1.0 or 2.0 grams of dry *M. albus* colonized rye seed were placed on filter paper in Petri dish lids on top of a stack of seven PDA dishes containing the test pathogen species’ conidia in each jar, as described above. The jar lids were sealed air tight with putty and SDW equal to the weight of *M. albus*-colonized rye seed was injected onto the filter through the rubber septum with a syringe. The non-treated water control received 2 mL of SDW injected onto the filter paper. The addition of water activated the *M. albus,* and volatile production occurred within the next 24 h. The jars and conidia cultures were incubated, and the number of germinated conidia was determined as described above.

### 3.2. Volatiles

In a second experiment, two active fungistatic/fungicidal volatiles, 2-methyl-1-butanol (2MB) and isobutyric acid (IBA), as determined in previous experiments [7], were tested individually for antifungal activity against toxigenic fungi. The same procedure was used as described above for testing the efficacy of *M. albus,* except in this experiment, *M. albus* was replaced with 25, 50, 75 or 100 μL/L of 2MB or 12.5, 25.0, 37.5 or 50.0 μL/L of IBA applied to the filter paper immediately before sealing the jars. The control treatments used 100 μL/L of SDW in place of the volatiles. Petri dishes of fungal conidia, freshly-seeded onto PDA, were exposed to the volatiles for 24, 48 or 72 h at 20 °C. Treated conidial cultures were incubated, and conidial germination was determined as before.

In earlier papers, Ezra and Strobel [14] and Mercier and Jiménez [1] identified one of the main *M. albus* volatiles as 2-methyl-1-butanol, but papers after Ezra *et al.*[4] listed 3-methyl-1-butanol as a *M. albus* volatile, but not 2-methyl-1-butanol. To determine if there was a difference in antimicrobial activity between 2MB and 3MB, the two volatiles were compared following the procedure in the second experiment detailed above using conidia of the same seven toxigenic fungi and the same concentrations of volatiles and incubation conditions described above.

In a third experiment, mixtures of 2MB and IBA were prepared such that 25 μL/L 2MB plus 12.5 μL/L IBA, 50 μL/L 2MB plus 25 μL/L IBA, 75 μL/L 2MB plus 37.5 μL/L IBA, 100 μL/L 2MB plus 50 μL/L IBA or 100 μL/L water (control) were applied to the filter papers as before. Petri dishes seeded with fungal conidia were removed from the jars after 48 or 72 h and incubated until a total of seven days of incubation had been completed. Conidial germination was determined as before.

Synergism was determined based on the Gowing formula described by Kosman and Cohen [12]. Two pesticides are considered synergistic when the observed effect divided by the expected effect is greater than one (*R* = *C*_obs_/*C*_exp_). The expected effect (*C*_exp_) of a mixture of pesticides is calculated as follows: *C*_exp_ = *C*_2_ + *C*_1_(1 − *C*_2_), where *C*_1_ and *C*_2_ are the observed effects for pesticide 1 and pesticide 2 when applied individually. The individual effects of volatiles in experiment 2 were used to calculate *C*_exp_, and the efficacy values from experiment 3 were the *C*_obs_ values used to determine the synergism ratio *R*; where *R* > 1.0 is indicative of synergism, *R* < 1.0 is indicative of antagonism and *R* = 1.0 is indicative of an additive effect.

In a fourth experiment, the same procedure was followed as in the third experiment, except the jars were sealed before the volatiles were applied to the filter paper. The jars were purged with either low oxygen air (1% O_2_ plus 0.03% CO_2_) or high CO_2_ air (20.8% O_2_ plus 15% CO_2_). An ambient air control (20.8% O_2_ plus 0.03% CO_2_) with water replacing the volatiles was utilized to determine if the atmospheres themselves would kill/suppress fungal conidia. Once the controlled atmospheres (CA) had been established in the jars (about 30 min) the volatile mixtures or water (control) were injected onto the filter papers with a syringe through the rubber septum. The jars were then incubated for 72 h at 3 °C. Finally, the Petri dishes were removed from the jars and incubated for seven days at 20 °C, and the numbers of colonies arising from germinated conidia were recorded as before.

### 3.3. Statistics

Data on the proportion of conidia killed/suppressed (dead or non-germinated conidia/total No. of conidia counted) constituted binomial data and was angular transformed prior to analysis. Data was analyzed with Genstat v14.1 (VSN International Ltd., Harpenden, Hertfordshire, UK, 2011) using a randomized complete block design with replication over time. Where angular transformation did not improve the analysis of variance, analysis of the non-transformed proportional data was reported for ease of presentation and interpretation.

## 4. Conclusions

*Muscodor albus* initially appeared to have significant potential as a biofumigant with the ability to control a wide range of pests and pathogens. However, as a living organism from a tropical climate, its abilities to function at temperatures below 5 °C are reduced and limit its efficacy in some applications. In addition, it has been recently reported that a previously unknown volatile produced by *M. albus* raises concerns for human toxicity, and registration of this product has been suspended pending further testing and investigation [15–17]. Two major volatiles of *M. albus*, isobutyric acid and 2- or 3-methyl-1-butanol demonstrated significant antifungal activity. Mixtures of the two volatiles were efficacious in killing/suppressing the conidia of seven mycotoxin-producing fungi *in vitro* under ambient air conditions and controlled atmosphere storage at 3 °C. These volatiles may be an acceptable alternative to *M. albus* as a biofumigant without the limitations and issues surrounding the use of a living organism.

## Figures and Tables

**Table 1:** Efficacy of *Muscodor albus*-produced volatiles in preventing germination and growth of mycotoxin-producing *Aspergillus*, *Penicillium* and *Fusarium* species *in vitro*. Freshly-sown conidia on potato dextrose agar were exposed to volatiles for 24 h at 20 °C in sealed jars and then incubated for six days before determining the proportion of conidia killed or suppressed (dead or non-germinated conidia/total No. of conidia). Analysis of variance was conducted on angular transformed and non-transformed data. Transformation did not improve means separation, and therefore, the non-transformed proportion of conidia killed or suppressed from germinating is reported below.

Fungal species								
*M. albus* g/L	*A. carbonarius*	*A. flavus*	*A. niger*	*A. ochraceus*	*P. verrucosum*	*F. culmorum*	*F. graminearum*	Mean
0	0.00	0.00	0.00	0.00	0.00	0.00	0.00	0.00
0.5	1.00	0.995	0.995	0.998	0.82	1.00	0.364	0.88
1.0	1.00	1.00	1.00	1.00	0.988	1.00	1.00	1.00
2.0	1.00	1.00	1.00	1.00	1.00	1.00	1.00	1.00
Mean	0.75	0.75	0.75	0.75	0.70	0.75	0.59	

Main Effects		Fungal species			df = 81	se = 0.01	*p* < 0.001	
		*M. albus* rate			df = 81	se = 0.01	*p* < 0.001	
Interaction		Species × Rate			df = 81	se = 0.02	*p* < 0.001	

**Table 2:** Efficacy of two main *Muscodor albus* volatile components, isobutyric acid (IBA) and 2-methyl-1-butanol (2MB), in preventing germination of mycotoxin-producing *Aspergillus*, *Penicillium* and *Fusarium* species *in vitro*. Freshly-sown conidia on potato dextrose agar were exposed to *M. albus* volatiles for 24, 48 and 72 h at 20 °C in sealed jars and then incubated in ambient air for seven days before determining the proportion of conidia killed/suppressed (dead or non-germinated conidia/total No. of conidia). Only the main effect means and 72 h exposure values for the fungal species × volatile × concentration interaction are presented. Analysis of variance was conducted on angular transformed and non-transformed data. Transformation did not improve means separation, and therefore, the non-transformed proportion of conidia killed/suppressed is reported below.

Fungal species							

Volatile Conc. μL/L	*A. carbonarius*	*A. flavus*	*A. niger*	*A. ochraceus*	*P. verrucosum*	*F. culmorum*	*F. graminearum*
**IBA**							

0	0.0	0.0	0.0	0.0	0.0	0.0	0.0
12.5	0.0	0.11	0.15	0.09	0.11	0.24	0.38
25.0	0.05	0.22	0.26	0.32	0.05	0.90	0.69
37.5	0.10	0.41	0.37	0.37	0.50	1.00	1.00
50.0	0.76	0.38	0.29	0.48	0.82	1.00	1.00

**2MB**							

0	0.0	0.0	0.16	0.0	0.0	0.0	0.0
25	0.09	0.14	0.18	0.43	0.1	0.07	0.15
50	0.05	0.15	0.22	0.45	0.03	0.11	0.15
75	0.47	0.27	0.68	0.32	0.30	0.06	0.08
100	0.82	0.70	0.87	0.62	0.69	0.11	0.26
**Mean**							
**Fungi**	0.23	0.25	0.32	0.31	0.26	0.35	0.37

**Exposure Time**		**24 h**	**48 h**	**72 h**			
		0.20	0.32	0.37			

**Volatile type**		**IBA**	**2MB**				
		0.34	0.25				

**Concentration**		**0**	**1×**	**2×**	**3×**	**4×**	
		0.01	0.16	0.26	0.43	0.63	

**Main Effects**							
Fungal spp.			df = 256	se = 0.03	*p* = 0.011		
Exposure time			df = 256	se = 0.02	*p* < 0.001		
Volatile type			df = 256	se = 0.02	*p* < 0.001		
Concentration			df = 256	se = 0.03	*p* < 0.001		
**Interaction**							
Species × Time			df = 256	se = 0.05	*p* = 0.026		
Species × Volatile			df = 256	se = 0.04	*p* < 0.001		
Time × Volatile			df = 256	se = 0.03	*p* = 0.85		
Species × Conc.			df = 256	se = 0.07	*p* < 0.001		
Time × Conc.			df = 256	se = 0.05	*p* = 0.01		
Volatile × Conc.			df = 256	se = 0.04	*p* = 0.002		

**Table 3:** Efficacy of combining two main *Muscodor albus* volatile components, isobutyric acid (IBA) and 2-methyl-1-butanol (2MB), in preventing germination and growth of mycotoxin-producing *Aspergillus*, *Penicillium* and *Fusarium* species *in vitro*. Freshly-sown conidia on potato dextrose agar were exposed to IBA plus 2MB for 48 h or 72 h at 20 °C in sealed jars and then incubated for seven days before determining the proportion of conidia killed/suppressed (dead or non-germinated conidia/total No. of conidia). Only the values for the volatile concentration and pathogen main effects and pathogen × volatile interaction are presented. Analysis of variance was conducted on angular transformed and non-transformed data. Transformation did not improve means separation, and therefore, the non-transformed proportions of conidia killed/suppressed by a treatment are reported below.

Volatile Conc. IBA/2MB μL/L	Fungal species							Mean

	*A. carbonarius*	*A. flavus*	*A. niger*	*A. ochraceus*	*P. verrucosum*	*F. culmorum*	*F. graminearum*	
0/0	0.00	0.00	0.00	0.00	0.00	0.00	0.00	**0.00**
12.5/25	0.020	0.01	0.29	0.13	0.10	0.00	0.27	**0.14**
25/50	0.22	0.10	0.05	0.24	0.64	0.99	0.49	**0.39**
37.5/75	0.46	0.67	0.77	0.59	0.88	0.81	0.75	**0.70**
50/100	1.00	0.82	0.90	1.00	1.00	1.00	0.96	**0.95**

**Mean**	**0.38**	**0.32**	**0.40**	**0.39**	**0.52**	**0.56**	**0.49**	

**Main Effects**		Species			df = 69	se = 0.05	*p* = 0.017	
		Exposure time			df = 69	se = 0.03	*p* = 0.174	
		Concentration			df = 69	se = 0.04	*p* <0.001	
**Interaction**		Species × Time			df = 69	se = 0.07	*p* = 0.162	
		Species × Conc.			df = 69	se = 0.12	*p* = 0.011	
		Time × Conc.			df = 69	se = 0.07	*p* = 0.837	
		Spp. × Time × Conc.			df = 69	se = 0.16	*p* = 0.741	

**Table 4:** Analysis of variance results of the proportion of fungal conidia killed/suppressed by four concentrations of isobutyric acid plus 2-methyl-1-butanol (25 + 50, 50 + 100, 75 + 150 or 100 + 200 μL/L of container headspace). Simultaneously, the fumigant treated conidia on potato dextrose agar in sealed glass jars were exposed to low oxygen (1% O_2_ and 0.03% CO_2_) or high CO_2_ (20.8% O_2_ and 15% CO_2_) atmospheres or ambient air (20.8% O_2_ and 0.03% CO_2_) at 3 °C for 72 h. Proportion of conidia killed/suppressed (dead or non-germinated conidia/total No. of conidia) by treatments was determined after seven days of post-treatment incubation. Only the values for the pathogen and volatile concentration main effects are presented. No interactions were statistically significant (*p* = 0.05). Proportion of conidia killed/suppressed was angular transformed prior to statistical analysis, and the back-transformed proportions appear in brackets.

Fungal species						

*A. carbonarius*	*A. flavus*	*A. niger*	*A. ochraceus*	*P. verrucosum*	*F. culmorum*	*F. graminearum*
3.81 (0.44)	3.34 (0.34)	3.29 (0.33)	3.02 (0.28)	4.01 (0.49)	3.83 (0.45)	3.83 (0.45)

		**Volatile Concentration (IBA/2MB, μL/L)**				
		
0/0	12.5/25	25/50	37.5/75	50/100		
0.36 (0.004)	1.94 (0.11)	4.46 (0.61)	5.56 (0.90)	5.74 (1.00)		

**Main Effects**	Species		df = 69	se = 0.20	*p* = 0.005	
	Atmosphere		df = 69	se = 0.11	*p* = 0.679	
	Concentration		df = 69	se = 0.17	*p* < 0.001	
